# Haplotype data and forensic evaluation of 23 Y-STR and 12 X-STR loci in eight ethnic groups from Eritrea

**DOI:** 10.1007/s00414-020-02446-2

**Published:** 2020-10-22

**Authors:** Carla Bini, Stefania Sarno, Elisabetta Tangorra, Alessandra Iuvaro, Sara De Fanti, Yohannes Ghebremedhin Tseghereda, Susi Pelotti, Donata Luiselli

**Affiliations:** 1 Department of Medical and Surgical Sciences, Unit of Legal Medicine, University of Bologna, via Irnerio, 49, 40126 Bologna, Italy; 2 Department of Biological, Geological and Environmental Sciences-Lab. of Molecular Anthropology & Centre for Genome Biology, University of Bologna, Via Selmi 3, 40126 Bologna, Italy; 3 Interdepartmental Centre “Alma Mater Research Institute on Global Challenges and Climate Change (Alma Climate)”, University of Bologna, Bologna, Italy; 4 Jomo Kenyatta University and Agriculture and Technology (JKUAT), P.O. Box 62 000, Nairobi, 00200 Kenya; 5 Department of Cultural Heritage, Ravenna Campus, University of Bologna, Via degli Ariani 1, 48121 Ravenna, Italy

**Keywords:** Y-STRs, X-STRs, Eritrean ethnic groups, Forensic parameters, Population genetics

## Abstract

Eritrea is a multi-ethnic country of over 3 million of people consisting of different ethnic groups, having each its own language and cultural tradition. Due to the lack of population genetic data for markers of forensic interest, in this study, we analyzed the genetic polymorphisms of 23 Y-chromosome STR loci and of 12 X-chromosome STR loci in a sample of 255 unrelated individuals from 8 Eritrean ethnic groups, with the aim to generate a reference haplotype database for anthropological and forensic applications. X- and Y-chromosomes markers may indeed offer information especially in personal identification and kinship testing, when relying on the availability of large local population data to derive sufficiently accurate frequency estimates. The population genetic analyses in the Eritrean sample for both the two set of Y- and X-STR markers showed high power of discrimination both at country-based and population levels. Comparison population results highlight the importance of considering the ethnic composition within the analyzed country and the necessity of increasing available data especially when referring to heterogeneous populations such as the African ones.

**Electronic supplementary material:**

The online version of this article (10.1007/s00414-020-02446-2) contains supplementary material, which is available to authorized users.

Eritrea is a multi-ethnic country located in the Horn of Africa with nine officially recognized ethnic groups in its population of over 3 million people [1]. Between Afro-Asiatic communities, Tigrinya make up about 55% of the population, and the Tigre constitute around 30% of the residents, whereas Rashaida are one of the smallest ethnic groups representing ~ 2% of the population. Most of the rest of the population belong to the other Afro-Asiatic-speaking communities of the Cushitic branch, such as the Afar, Bilen, Hedareb, and Saho. The Kunama and Nara populations represent small Nilo-Saharan ethnic groups in the country [2].

Population genetic data for markers of forensic interest are still limited for some populations, in particular of African origin. As concern the Eritrean population, a previous study on the Y-chromosome variability in East-Africa countries analyzed a total of 161 individuals belonging to 5 different ethnic groups of Eritrea [3], while, to the best of our knowledge, no data are available for X-chromosome markers. X- and Y-chromosomes markers may provide useful information especially in personal identification and kinship testing, when relying on the availability of large local population data to derive sufficiently accurate frequency estimates.

With the aim to generate a relevant reference database for anthropological and forensic statistical evaluations, we analyzed the genetic polymorphisms of 23 Y-chromosome STR loci and of 12 X-chromosome STR loci in a sample of 255 unrelated individuals from 8 Eritrean ethnic groups. In particular, the Eritrean dataset was sampled based on ethno-linguistic information and consists of Afar-Cushitic (*n* = 21), Bilen-Cushitic (*n* = 15), Hedareb-Cushitic (*n* = 15), Kunama Nilo-Saharan (*n* = 12), Nara Nilo-Saharan (*n* = 33), Saho-Cushitic (*n* = 21), Tigre-Semitic (*n* = 62), and Tigrinya-Semitic (*n* = 76) groups.

DNA anonymous samples had been stored in the laboratory without any individual associated information, and the markers analyzed in this study identify only the geographical distribution of genetic lineages. The present research was approved by the Bioethical Committee of the University of Bologna.

All samples were amplified for the 23 Y-STRs included in the PowerPlex® Y23 System (Promega) as well as for the 12 X-STRs loci included in the Investigator Argus X-12 kit (Qiagen), following the manufacturer recommended protocols. PCR products were separated and detected on an ABI PRISM 310 Genetic Analyzer using POP-4 polymer; alleles were called and binned by GeneMapper ID v3.2 software (Thermo Fisher Scientific) according to the manufacturer’s instructions.

Haplotype data were submitted to the Y-chromosomal Haplotype Reference Database (YHRD, www.yhrd.org) [4], and the following accession numbers were assigned: YA004649 for Afar, YA004650 for Bilen, YA004651 for Hedareb, YA004652 for Saho, YA004653 for Kunama, YA004654 for Nara, YA004655 for Tigre, and YA004656 for Tigrinyas.

Arlequin v3.5.1.3 software [5] was used to calculate Y-chromosome standard diversity index, haplotype diversity (HD), average gene diversity over loci, and mean number of pairwise differences, and the forensic parameters, match probability (MP) and discrimination capacity (DC). Pairwise genetic distances (*Rst* values) based on Y-chromosome STR data were calculated between the Eritrean population and a wide set of reference comparison groups [6] and used to generate a multidimensional scaling (MDS) plot with the software R (library MASS) [7].

For X-chromosome loci, allele frequencies, haplotype frequencies for each linkage group (LG), and forensic efficiency parameters (gene diversity, GD; polymorphism information content, PIC; power of discrimination, PD; mean exclusion chance, MEC) were generated using the StatsX v2.0 software as described in [8]. Linkage disequilibrium (LD) in the male population sample was estimated by using the Arlequin software [5]. Inter-population Fst genetic distances based on haplotype frequencies between the Eritrean dataset and 13 comparison populations from Europe, Asia, and Africa [9–18] were integrated for each of the four X-STRs linkage groups and graphically represented by using the R software package DISTATIS [19].

In our population sample, we found 238 different Y-haplotypes, of which 221 were unique, with the other 17 instead shared by pairs of two subjects (Table S1). Excepting a single case of sharing between Hedareb and Kunama populations, all the subjects that shared the same haplotype also belonged to the same ethnic group. Allele frequencies for the 255 Eritrean samples are reported in Table S2. One null allele at the DYS19 locus and one duplication at the DYS439 locus were observed in two different individuals belonging respectively to the Nara and Tigre ethnic groups. The microvariant alleles 15.2, 17.2, 18.2, 19.2, 20.2, and 21.2, not included in the bin set of the allelic ladder, were observed at locus DYS458 in twenty-nine samples. The microvariant allele 17.1 at locus DYS385a/b and the rare variant allele 4 at locus DYS643 were respectively found in two different samples from Tigre. All these variants are already reported in locus information on YHRD [4].

Diversity indexes and forensic parameters for the PPY23 markers set are shown in Table S3. Overall, the typing of Y-chromosome loci revealed low cases of haplotype sharing, resulting in relative high values of haplotype diversity and good discrimination capacity. At a population level, the Eritrean ethnic group that showed the lower values of both HD and DC was the Cushitic-speaking population of Saho, as already reported in a previous study [3].

The data for the Eritrea population newly reported in the present study were compared with those of other 20 Eastern African population ethnic groups from Eritrea, Ethiopia, Djibouti, and Kenya already available from the literature. When compared by using all the 21 Y-STRs loci common to both studies, the considered countries showed significant *Rst* genetic distances among each other, thus confirming the presence of a notable genetic sub-structure within East Africa as described previously [3]. Importantly, significant *Rst* values were observed not only at a country-based level but also at a population-level when considering pairwise comparisons among single ethnic groups (Table S4). While not excluding different sample sizes and sampling strategies, part of the differences observed between the Eritrean samples presented by this and the previous study could however be driven by the outlying genetic composition of the Saho ethic group. Indeed, the genetic differences at a country-based level between the two Eritrean population samples become close to zero and not significant once excluding this ethnic group from the comparison. Accordingly, when we directly compared the haplotype composition of single ethnic groups which were sampled in both of the studies, significant genetic differences were found among the two Saho samples, but not in the comparisons between the two Kunama, the two Nara or the two Tigre collected populations. This may be related to the peculiar genetic composition already described for the Saho sample [3], that was indeed shown to be characterized by the lowest values of intra-population diversity parameters and by high frequencies (~ 88%) of a peculiar Y-chromosome haplogroup (E-V22) which is relatively uncommon in the other African populations.

A MDS analysis was further performed on the linearized *Rst* genetic distances calculated by considering the Eritrean population and 77 comparison groups from Africa, Europe, and the Middle East for which a comparable level of analysis for all the 23 Y-chromosome STR loci was available [6]. The first MDS dimension clearly separates the African groups from the non-African ones (Fig. S1). Along this axis of variation, the Eritrean population particularly clusters with the East African Kenyan Maasai, being located in an intermediate position between populations from Central and South Africa (Yoruba, Zimbabwe, and Xhosa) on one hand and those from the Middle East and South-Eastern Europe on the other hand. A North-to-South gradient of genetic variation finally characterizes the pattern observed within Europe along the second MDS dimension.

As concern X-STR results, twenty-two out of the 255 analyzed males showing a locus dropout—of which 16 samples for the DXS10148 and 6 for the DXS10146 locus, respectively—were excluded from statistical analyses. Presumably, these silent alleles are due to one or more mutations in the primer binding sites, which reduce the efficiency of the PCR reaction, as previously observed for the African populations [11, 14]. Indeed, amplification performed with higher DNA concentration showed small off-ladder peaks in DXS10148 system that need to be further investigated, by also considering the new sequence variant identified for this locus [20]. Furthermore, a total of 32 off-ladder alleles in the linkage groups I, II, and IV were detected and designated according to their base pair sizes (Table S5). Overall, the allele and haplotype frequencies for the X-STRs loci and corresponding linkage groups (LGs) are detailed in Table S6 and Table S7, respectively.

The number of observed haplotypes and the forensic parameters calculated for each of the four LGs are reported in Table S8. The most informative linkage group is LG1 (PIC = 0.9936) including the DXS10135 marker that showed the highest forensic efficiency (PIC = 0.94) with 30 different alleles typed. The least polymorphic locus was instead the DXS7423 counting for only 5 alleles. As expected, evidence of significant linkage disequilibrium (LD) was found between markers within the same LG, but significant associations were also observed between loci of LG2/LG4 and LG3/LG4 groups and more precisely for DXS10135-DXS7423 and for DXS10079-DXS10146 pairs (Table S9) probably related to the population history, such as the presence of population sub-structure, non-random mating, or local genetic drift. Haplotype frequencies for the four X-chromosome linkage groups were finally used to summarize the relationships between Eritreans and other populations from Europe, Asia, and Africa. The DISTATIS plot in Fig. S2 generally shows a pattern of genetic structuring that overall resembles the geographic distribution of the considered populations according to their continent of origin, placing the Eritreans between European and North-African populations on one hand and the other West-African groups on the other and thus reflecting the pattern also shown by PPY23 system analysis.

In conclusion, the present study provides a contribution for population databasing, adding new allele and haplotype frequency estimates at both Y-STR and X-STR loci from a population not easily accessible for sampling. The typing of 255 male individuals largely extends previous Y-chromosome STRs data available for the Eritrean population and constitutes a first reference dataset for X-chromosome STRs useful for statistical evaluation in forensic casework. Overall, our results remark the importance of implementing population databases by including representative data from local populations, especially when referring to heterogeneous groups such as the African ones.

This manuscript follows the recommendations of the ISFG on the use of YSTRs and X-STRs in forensic analysis [21, 22] and the guidelines for publication of population data requested by the journal [23].

## Electronic supplementary material

ESM 1 (XLSX 78 kb)

ESM 2 (PDF 74 kb)

ESM 3 (PDF 61 kb)
