# Supplementary material for: Haplotype data and forensic evaluation of 23 Y-STR and 12 X-STR loci in eight ethnic groups from Eritrea
Source: Int J Legal Med. 2020 Oct 22;135(2):449–53. doi: 10.1007/s00414-020-02446-2 (PMC7870587; doi:10.1007/s00414-020-02446-2)

Fig. S1 Two-dimensional MDS plot based on Y-chromosome data of analyzed Eritrean and 77 comparison population groups. Population labels are color-coded based on the macro-geographic area as specified in the legend at the top-right

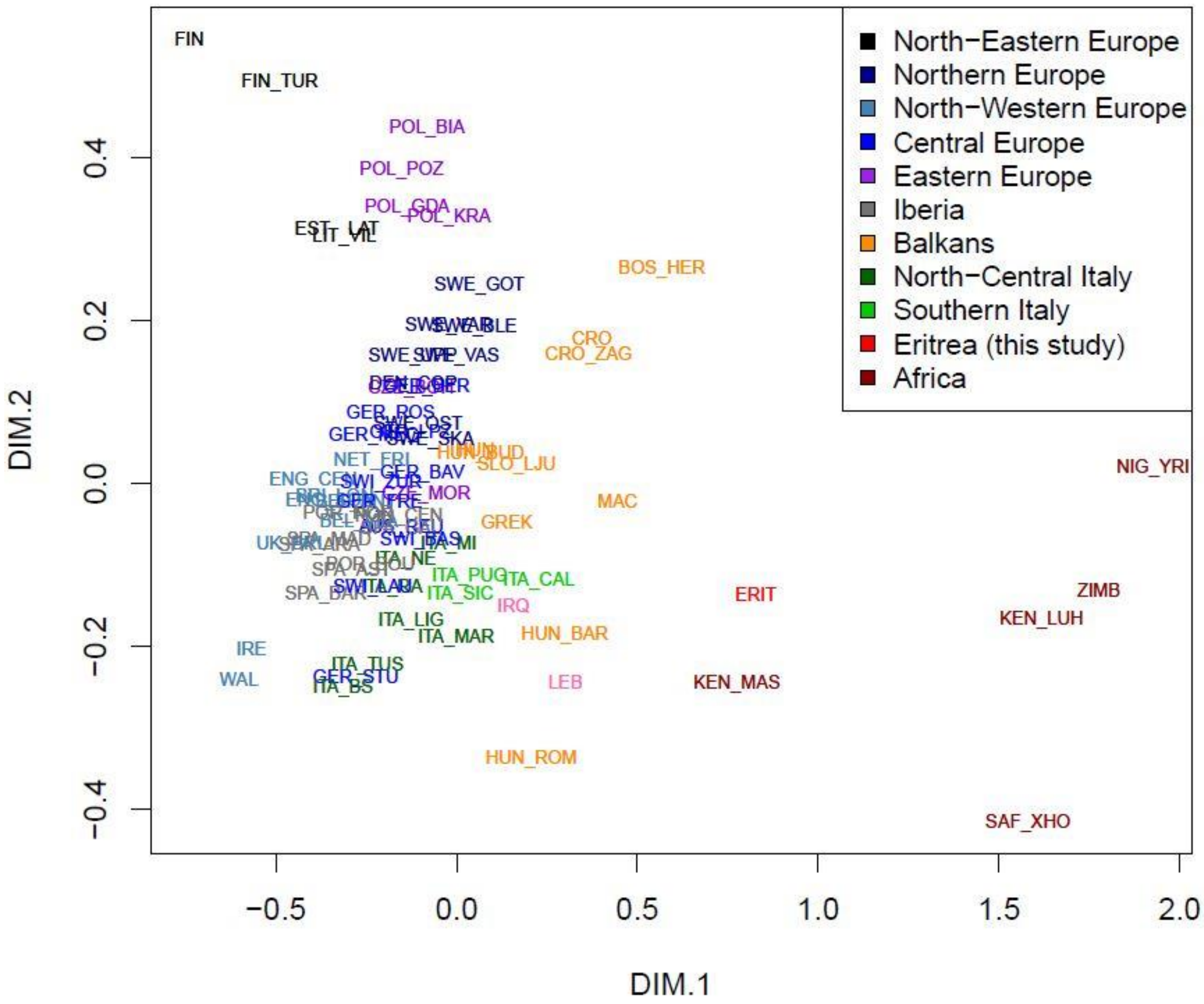

Supplement: Supplementary file 2 — (PDF 74 kb) [file 414_2020_2446_MOESM2_ESM.pdf]
