# Supplementary material for: Haplotype data and forensic evaluation of 23 Y-STR and 12 X-STR loci in eight ethnic groups from Eritrea
Source: Int J Legal Med. 2020 Oct 22;135(2):449–53. doi: 10.1007/s00414-020-02446-2 (PMC7870587; doi:10.1007/s00414-020-02446-2)

Fig. 2 DISTATIS analysis based on X-haplotype frequencies for the four linkage groups in 13 populations from Africa, Asia and Europe

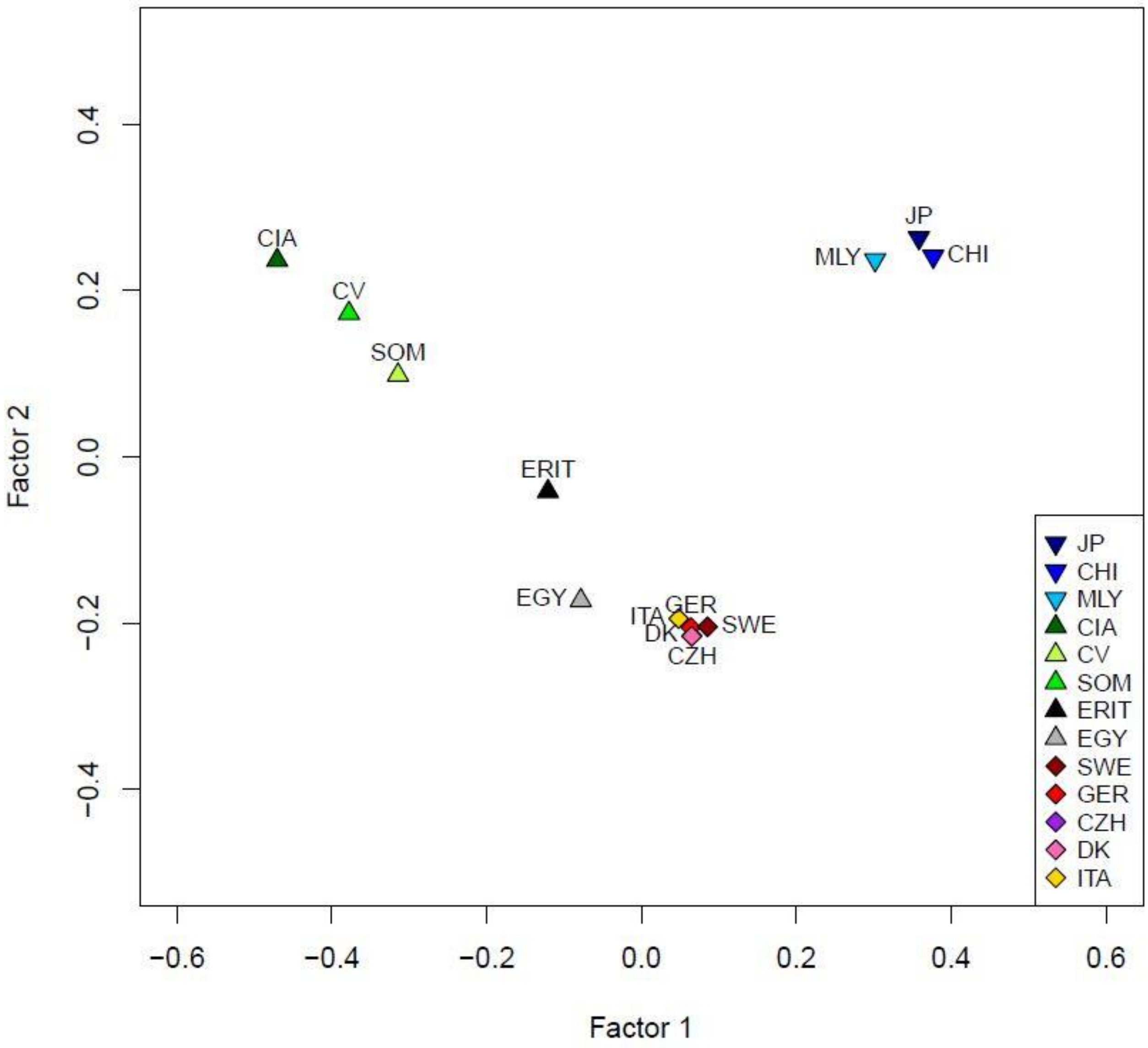

Supplement: Supplementary file 3 — (PDF 61 kb) [file 414_2020_2446_MOESM3_ESM.pdf]
